# Impact of Hypoalbuminemia on Morbidity and Mortality After Radical Cystectomy

**DOI:** 10.3390/cancers18020313

**Published:** 2026-01-20

**Authors:** Sri Saran Manivasagam, Jay D. Raman, Matthew G. Kaag

**Affiliations:** Department of Urology, Penn State Milton S. Hershey Medical Center, 500 University Dr, Hershey, PA 17033, USA; smanivasagam@pennstatehealth.psu.edu (S.S.M.); jraman@pennstatehealth.psu.edu (J.D.R.)

**Keywords:** bladder cancer, radical cystectomy, albumin, 30-day mortality, morbidity, nutrition

## Abstract

Objective: To evaluate the role of serum albumin in predicting surgical outcomes after radical cystectomy. Methods: Retrospective cohort analysis of adults who underwent radical cystectomy from 2019 to 2022 within the ACS NSQIP dataset. Patients were stratified into three groups based on preoperative serum albumin: <3.0 g/dL, 3.0–3.5 g/dL, and >3.5 g/dL. Primary outcomes were surgical site infections, wound disruption, reoperation rates, prolonged hospitalization, and 30-day mortality. Regression analyses assessed the impact of hypoalbuminemia (<3.5 g/dL) on outcomes. Results: In total, 6748 patients were included in the analysis. Distribution of preoperative albumin levels included 4.8% with albumin less than 3.0 gm/dL (Cohort A), 10.7% with albumin between 3.0 and 3.5 gm/dL (Cohort B), and 84.4% with albumin > 3.5 gm/dL (Cohort C). The incidence of superficial SSI (7.7%), deep SSI (2.2%), wound disruption (4.0%), unplanned reoperation (8.6%), and still in hospital at 30 days (5.5%) was significantly higher in Cohort A (*p* for all < 0.05). Moreover, 30-day mortality was significantly higher in Cohorts A and B, as compared to Cohort C (2.2% vs. 2.3% vs. 1.3%, *p* = 0.03). On multivariate logistic regression, albumin < 3.5 gm/dL was significantly associated with reoperation (OR = 1.39, *p* = 0.031), prolonged hospitalization (OR = 1.28, *p* = 0.038), and 30-day mortality (OR = 1.74, *p* = 0.025). Conclusions: Preoperative hypoalbuminemia is independently associated with increased morbidity and mortality following radical cystectomy. Given its modifiable nature, serum albumin should be considered a key target for preoperative optimization.

## Simple Summary

Radical cystectomy is a major surgery for bladder cancer, but it carries high risks of complications and death. Poor nutrition before surgery can worsen these risks. Serum albumin, a simple blood test, reflects the nutritional status and may predict outcomes. In this study, we analyzed thousands of patients to see how albumin levels relate to infections, reoperations, the hospital stay, and survival. We found that even small drops in albumin significantly increase complications and mortality. Because albumin is easy to measure and can be improved before surgery, these findings highlight the importance of nutritional optimization. This research may guide surgeons and care teams to include albumin-based screening and prehabilitation in treatment plans, improving recovery and reducing risks for patients undergoing bladder cancer surgery.

## 1. Introduction

Bladder cancer is the tenth most common cancer worldwide, with an annual incidence of 573,278 cases, and has a four-fold higher incidence in men than women [1]. Radical cystectomy (RC) remains the mainstay of treatment for muscle-invasive bladder cancer and is also increasingly recommended for BCG refractory or unresponsive high-risk non-muscle invasive bladder cancer. Over 10,000 such surgeries are performed annually in the United States. Irrespective of the surgical approach, RC is associated with a relatively high morbidity, with infectious complications and ileus being the most common [2]. Such complications contribute to a longer hospital duration and greater hospital costs [3]. Furthermore, the morbidity of RC is associated with 20–30% re-admission rates, and 20% of all patients require further invasive procedures [4,5,6,7].

Malnutrition is a prevalent problem in patients undergoing RC. In general, preoperative malnutrition contributes to increased rates of postoperative complications. Nutritional optimization prior to surgery shows significant potential for improved outcomes [8,9,10]. Other conditions predisposing to hypoalbuminemia are alcohol use disorder, cirrhosis, diabetes, heart failure, infections, inflammatory bowel disease, nephrotic syndrome, liver disease, systemic lupus erythematosus, and thyroid pathology [11,12,13]. Multiple markers of the nutritional status and frailty have been studied, such as the prognostic nutritional index (PNI), the Controlling Nutritional Status Score, and the Psoas Muscle Index. Collectively, these indices have been found to correlate significantly with surgical outcomes after RC [14,15,16,17]. Nonetheless, assessment of these measures can be labor intensive, thereby potentially limiting clinical utility in busy surgical practices.

In contrast, serum albumin is a simple, readily available marker that has been found to correlate with the length of stay and mortality after RC [18,19,20]. However, the association of albumin levels and morbidity RC has not been widely reported. Furthermore, these results are limited in their small sample sizes and single-center study nature. By understanding the association of serum albumin with such complications and the optimal method of nutritional rehabilitation prior to surgery, we can improve outcomes after RC.

Large-scale, multi-institutional studies that characterize the risk gradient across specific albumin thresholds and its impact on a comprehensive set of morbidities are limited. Here then, we aimed to evaluate the impact of serum albumin on morbidity and mortality after radical cystectomy. We utilized the American College of Surgeons National Surgical Quality Improvement Program database (ACS NSQIP) and ACS procedure targeted database of cystectomy from 2019 to 2022 to identify patients who underwent radical cystectomy. We aimed to further understand the role of albumin in complications by building three cohorts of patients: those with albumin at less than 3 gm/dL, those with albumin at between 3 and 3.5 gm/dL, and those with albumin at more than 3.5 gm/dL.

## 2. Materials and Methods

### 2.1. Study Cohort and Design

The ACS NSQIP database and procedure targeted cystectomy database from 2019 to 2022 identified RC cases. The ACS NSQIP collects data on over 150 variables, including preoperative risk factors, intraoperative variables, and 30-day postoperative morbidity and mortality outcomes for patients undergoing major surgical procedures in both the inpatient and outpatient settings. Patients aged 18–90 years with the Current Procedure Terminology (CPT) code for cystectomy were included in the analysis. The American College of Surgeons National Surgical Quality Improvement Program and the hospitals participating in the ACS NSQIP are the sources of the data used herein; they have not verified and are not responsible for the statistical validity of the data analysis or the conclusions derived by the authors.

Only patients with complete data were included in the analysis. As such, 15% of cases were excluded due to missing albumin values. Included patients were divided into Cohorts A, B, and C, on the basis of preoperative serum albumin. Cohort A included patients with albumin at less than 3 gm/dL, Cohort B included patients with albumin at between 3 and 3.5 gm/dL, and Cohort C had patients with albumin at more than 3.5 gm/dL. This stratification was decided by the authorship team based on expert consensus. Patients in these cohorts were compared on the basis of demographic characteristics and comorbidity profiles. Furthermore, the incidence of complications such as superficial surgical site infection (SSI), deep SSI, organ-space SSI, wound disruption, sepsis, septic shock, unplanned reoperation, unplanned readmission, and mortality was compared across cohorts. Generative artificial intelligence was not used in the preparation of this manuscript.

### 2.2. Statistical Analysis

Patient demographics, clinical characteristics, and outcomes were evaluated using the chi-square test for categorical variables and one-way analysis of variance (ANOVA) for numerical variables. Univariate and multivariate logistic regression evaluated the association of hypoalbuminemia with outcomes such as reoperation rates, still in hospital after 30 days of surgery, and 30-day mortality. Variables used for regression were age > 60 years, BMI > 30 kg/m^2^, smoking, dependent functional status, diabetes mellitus (DM), chronic obstructive pulmonary disease (COPD), congestive heart failure (CHF), hypertension (HTN), chronic kidney disease (CKD) requiring dialysis, American Society of Anesthesiologists (ASA) classification system 3/4/5, operation time more than 300 min, and hypoalbuminemia (albumin less than 3.5 gm/dL). All models were tested for goodness-of-fit using the Hosmer–Lemeshow test and for multicollinearity using variance inflation factors. All tests were conducted using SPSS statistical software version 30, and *p*-values less than 0.05 were considered significant.

## 3. Results

A cohort of 6748 patients undergoing RC was identified. Table 1 summarizes baseline patient characteristics stratified across different cohorts. Overall, the mean age of patients was 70 years, with a mean BMI of 28 kg/m^2^. Differences between the cohorts were noted across several variables, including age, BMI, ASA score, diabetes, and dependent functional status. Conversely, the distribution of smokers, patients with severe chronic obstructive pulmonary disease (COPD), patients with congestive heart failure (CHF), and patients with hypertension (HTN) was similar across groups.

Table 2 highlights postoperative outcomes after RC stratified across the different cohorts. Notably, the incidence of superficial SSI (7.7% vs. 4.4% vs. 4.2%, *p* = 0.012) and deep SSI (2.2% vs. 0.7% vs. 0.5%, *p* = 0.001) was significantly higher in Cohort A compared to Cohorts B and C. However, the incidence of organ space SSI in each of the cohorts was not significantly different. The incidence of wound disruption was significantly higher in Cohort A (4%) and Cohort B (2.9%), as compared to Cohort C (1.5%) (*p* < 0.001). Unplanned reoperation rates (8.6% vs. 4.7% vs. 4.3%, *p* = 0.001) and still in hospital after 30 days of surgery rates (5.5% vs. 2.8% vs. 1.6%, *p* < 0.001) were significantly higher in Cohort A. The length of hospital stay (5.67 vs. 5.62 vs. 4.82 days, *p* < 0.001) was significantly longer in Cohorts A and B, as compared to Cohort C. Furthermore, 30-day mortality was significantly higher in Cohort B, followed by Cohorts A and C (2.3% vs. 2.2% vs. 1.6%, respectively, *p* = 0.035).

In the multivariate analysis, patients who were smokers (OR 1.59, 95% CI = 1.21–2.09, *p* < 0.001), diabetics (OR 1.43, 95% CI = 1.03–1.96, *p* = 0.028), patients with hypoalbuminemia (OR 1.39, 95% CI = 1.03–1.88, *p* = 0.031), and patients who were hypertensive (OR 1.36, 95% CI = 1.05–1.77, *p* = 0.02) had significantly greater odds of reoperation (Table 3). Furthermore, patients who were at a higher risk of staying in the hospital after 30 days of surgery were those who were hypertensive (OR 1.41, 95% CI = 1.12–1.87, *p* = 0.024), those with hypoalbuminemia (OR 1.28, 95% CI = 1.04–1.81, *p* = 0.038), and those with diabetes (OR 1.23, 95% CI = 1.04–1.86, *p* = 0.022) (Table 4).

Univariate logistic regression for the 30-day mortality outcome revealed that patients receiving dialysis (OR 5.22, 95% CI = 1.16–23.35, *p* = 0.031), a dependent status (OR 2.89, 95% CI = 1.14–7.33, *p* = 0.025), patients belonging to ASA3/4/5 (OR 2.36, 95% CI = 1.07–5.18, *p* = 0.032), and patients with albumin less than 3.5 (OR 1.83, 95% CI = 1.14–2.91, *p* = 0.011) were significantly associated with the outcome of interest. However, in multivariate analysis, only a dependent status (OR 2.61, 95% CI = 1.02–6.65, *p* = 0.044), patients belonging to ASA 3/4/5 (OR 2.31, 95% CI = 1.05–5.07, *p* = 0.036), and patients with albumin less than 3.5 gm/dL (OR 1.74, 95% CI = 1.07–2.83, *p* = 0.0025) were independently associated with 30-day mortality after radical cystectomy (Table 5).

## 4. Discussion

The primary finding of our study is that serum albumin is an important factor in both morbidity and mortality after radical cystectomy. In multivariate regression, hypoalbuminemia is significantly and independently associated with increased odds of return to OR, a hospital duration more than 30 days, and 30-day mortality. Serum albumin stands out in contrast to other factors implicated in morbidity and mortality after RC, as a risk factor that is potentially modifiable in the preoperative period between diagnosis and surgery.

Our study findings are consistent with, but also distinct in certain aspects from, prior studies on this particular topic. A study by Arora et al. in 2018, used the ACS NSQIP database, identified 2055 patients between 2006 and 2012 who underwent RC, and found that both hypoalbuminemia and obesity were associated independently with 30-day morbidity and 30-day mortality [21]. Another retrospective study, in 2014, observed 1097 RCs performed between 1992 and 2005, found the incidence of hypoalbuminemia to be 14%, and found a significant association with complications and 90-day mortality [20]. Similar results were observed by Caras et al., Bhalla et al., and Lambert et al.; they asserted the importance of albumin in major urologic oncological procedures [18,22,23].

However, our study stands out for a few reasons. No study has compared the outcomes across the three cohorts of patients based on serum albumin. This underscores the very important fact that even small variations in serum albumin to the tune of 0.5 gm/dL can have a clinically significant impact on outcomes. Furthermore, this study has a large sample size of 6748 patients, and the robustness of our univariate and multivariate logistic regression models is a salient feature. Prior to our analysis, the largest study among these was the study by Arora et al., which included 2055 patients.

Hypoalbuminemia is a prevalent problem in patients undergoing cancer surgery, especially those undergoing radical cystectomy [8,24]. The role of serum albumin as a surrogate marker of nutrition cannot be questioned, and the implications of hypoalbuminemia for morbidity and mortality have been well established in this study. However, the role of prehabilitation in improving the nutritional status prior to morbid procedures is less well studied. While albumin is a modifiable marker of the nutritional and inflammatory status, meaningful intervention is challenging due to the short timeframe between diagnosis and surgery for muscle-invasive bladder cancer [25]. More importantly, hypoalbuminemia may reflect underlying frailty, inflammation, or the disease burden rather than the nutritional status alone [26,27,28,29].

Roth et al. conducted a prospective randomized trial comparing enteral (n = 74) and parenteral nutrition (n = 83) in patients after radical cystectomy. They observed that parenteral nutrition after radical cystectomy is associated with a higher incidence of complications and higher costs, without translating to a shorter hospital stay or early bowel recovery [30] . In a prospective study of 1085 patients, in regard to preoperative nutrition, either 7 days of enteral or parenteral nutrition was associated with a shorter hospital stay only in patients who were malnourished [31] . More specifically, Hamilton-Reeves et al. performed a randomized, controlled trial comparing outcomes in patients who received special immune nutrition and those who received oral nutrition supplementation before and after radical cystectomy. Specialized immune nutrition was associated with a decreased postoperative complication rate by 33% and a lower incidence of infection [32] . At this time, the best approach to optimizing the nutritional status prior to any major surgery is not clearly established and is a potential avenue for research. In the era of enhanced recovery protocols, the area of preoperative nutrition has gained significant attention and is a common component of most enhanced recovery after surgery (ERAS) protocols [33] .

Despite the merits of our study, there are limitations that are important to consider. Around 15% of patients in the ACS NSQIP procedure targeted cystectomy database from 2019 to 2022 were excluded from the analysis due to missing preoperative serum albumin values. Even though albumin testing is relatively inexpensive, widely available, and significantly associated with outcomes, a significant fraction of patients do not have their serum albumin levels tested. The exclusion of such cases limits the generalizability of this study. Secondly, the retrospective nature of the study design is associated with inherent bias. However, conducting a study such as this one in a prospective manner with such a large cohort is a difficult task. Thirdly, the database does not differentiate between the types of urinary diversion, which may account for some bias. It is also important to note that the database captures only 30-day mortality and does not capture oncological outcomes or longer-duration outcomes. The lack of homogeneity in the distribution of patients with diabetes and those who are functionally independent across the three cohorts needs to be kept in mind, before drawing conclusions. In spite of these limitations, this study demonstrates the importance of serum albumin as a surrogate marker for nutrition and its role in predicting morbidity and mortality after radical cystectomy. This easily measured serum marker should be routinely assessed in bladder cancer patients and can serve as a barometer for intervention.

## 5. Conclusions

Serum albumin emerged as a powerful and potentially modifiable factor associated with morbidity and mortality after radical cystectomy, with even 0.5 g/dL decrements translating into prolonged hospitalization and greater rates of re-operation and mortality. These findings underscore the clinical imperative to measure albumin routinely and explore rapid, preoperative nutritional optimization within enhanced-recovery pathways. Future prospective studies are needed to define the most effective prehabilitation strategies and to validate albumin-guided interventions in this high-risk population.

## Figures and Tables

**Table 1:** Clinical and demographic characteristics.

	Cohort A (Albumin < 3 gm/dL) (*n* = 326)	Cohort B (Albumin 3–3.5 gm/dL) (*n* = 724)	Cohort C (Albumin > 3.5 gm/dL) (*n* = 5698)	All Cases (*n* = 6748)	*p*-Value
Mean age (yrs) (95% CI)	69.22 (68.17–70.26)	70.42 (69.71–71.13)	69.94 (69.71–70.17)	69.96 (69.74–70.17)	0.002
Mean BMI (kg/m^2^) (95% CI)	26.74 (26.08–27.40)	27.47 (27.04–27.90)	28.53 (28.37–28.68)	28.33 (28.19–28.47)	<0.001
ASA 3/4/5 (%)	86.5%	83.3%	82.7%	83.1%	<0.001
Smoker (%)	23.4%	23.2%	20.6%	21.0%	0.156
Diabetic (%)	18.5%	22.3%	21.3%	21.3%	0.017
Severe COPD (%)	7.4%	9.3%	7.3%	7.5%	0.167
CHF (%)	3.1%	4.0%	2.9%	3.0%	0.259
HTN (%)	59.1%	58.1%	61.4%	60.9%	0.189
Functional status: Independent (%)	93.2%	97.0%	98.5%	98.1%	<0.001

**Table 2:** Postoperative outcomes across cohorts A, B, and C.

	Cohort A Albumin < 3 gm/dL (*n* = 326)	Cohort B Albumin 3–3.5 gm/dL (*n* = 724)	Cohort C Albumin > 3.5 gm/dL (*n* = 5698)	All Cases (*n* = 6748)	*p*-Value
Occurrence of superficial SSI	7.7%	4.4%	4.2%	4.4%	0.012
Occurrence of deep SSI	2.2%	0.7%	0.5%	0.6%	0.001
Occurrence of organ space SSI	8.9%	7.5%	7.4%	7.5%	0.607
Occurrence of wound disruption	4.0%	2.9%	1.5%	1.8%	<0.001
Occurrence of sepsis	8.6%	7.0%	6.7%	6.8%	0.411
Occurrence of septic shock	3.1%	3.3%	2.5%	2.6%	0.405
Unplanned reoperation	8.6%	4.7%	4.3%	4.6%	0.001
Unplanned readmission	22.5%	19.6%	19.6%	19.7%	0.438
Still in hospital rates > 30 days	5.5%	2.8%	1.6%	1.9%	< 0.001
30-day mortality	2.2%	2.3%	1.3%	1.4%	0.035
Length of hospital stay	5.67 (4.32–7.02)	5.62 (5.25–5.95)	4.82 (1.92–7.72)	5.28 (4.42–5.65)	<0.001
Days from operation to wound disruption	10.54 (7.26–13.82)	11.43 (7.99–14.87)	12.22 (10.89–13.56)	11.90 (10.75–13.05)	0.376

**Table 3:** Univariate and multivariate logistic regression for re-operation.

Parameter	Univariate	*p*-Value	Multivariate	*p*-Value
Age > 60	1.046 (0.757–1.447)	0.783	1.008 (0.713–1.426)	0.962
BMI > 30	1.215 (0.946–1.561)	0.127	1.258 (0.972–1.630)	0.082
Smoking	1.561 (1.212–2.012)	<0.001	1.593 (1.214–2.090)	<0.001
Dependent status	1.055 (0.461–2.416)	0.899	1.085 (0.70–2.504)	0.849
Diabetes	1.284 (0.952–1.733)	0.102	1.430 (1.039–1.968)	0.028
COPD	1.459 (1.000–21.129)	0.050	1.215 (0.808–1.825)	0.349
CHF	1.199 (0.646–2.225)	0.566	1.196 (0.637–2.245)	0.578
HTN	1.291 (1.014–1.645)	0.038	1.367 (1.051–1.777)	0.020
Dialysis	1.408 (0.992–1.806)	0.742	1.210 (0.980–1.654)	0.988
ASA 3/4/5	1.080 (0.791–1.476)	0.627	1.013 (0.730–1.406)	0.938
Operation time > 300 min	1.155 (0.914–1.460)	0.226	1.095 (0.859–1.397)	0.463
Albumin less than 3.5 gm/dL	1.398 (1.045–1.854)	0.024	1.392 (1.030–1.881)	0.031

**Table 4:** Univariate and multivariate logistic regression for length of hospital stay more than 30 days.

Parameter	Univariate	*p*-Value	Multivariate	*p*-Value
Age > 60	1.610 (0.885–2.928)	0.119	1.024 (0.708–1.460)	0.904
BMI > 30	1.255 (0.865–1.823)	0.232	1.264 (0.868–1.308)	0.448
Smoking	1.042 (0.683–1.590)	0.849	1.298 (0.933–2.280)	0.566
Dependent status	3.161 (1.444–6.918)	0.004	1.084 (0.448–2.614)	0.804
DM	1.683 (1.154–2.456)	0.007	1.230 (1.049–1.860)	0.022
COPD	1.903 (1.133–3.195)	0.015	1.116 (0.878–1.625)	0.35
CHF	1.301 (0.526–3.216)	0.569	1.298 (0.686–2.440)	0.648
HTN	1.437 (0.986–2.093)	0.059	1.412 (1.122–1.875)	0.024
Dialysis	2.061 (0.277–15.324)	0.480	1.866 (0.656–13.985)	0.548
ASA 3/4/5	2.455 (1.284–4.696)	0.007	1.022 (0.756–1.464)	0.996
Operation time > 300 min	1.177 (0.823–1.683)	0.371	1.108 (0.844–1.386)	0.445
Albumin less than 3.5 gm/dL	2.316 (1.577–3.402)	<0.001	1.284 (1.045–1.810)	0.038

**Table 5:** Univariate and multivariate logistic regression for 30-day mortality.

Parameter	Univariate	*p*-Value	Multivariate	*p*-Value
Age > 60	1.270 (0.675–2.389)	0.458	1.005 (0.520–1.941)	0.520
BMI > 30	1.207 (0.746–1.953)	0.443	1.166 (0.720–1.889)	0.533
Smoking	1.470 (0.826–2.613)	0.190	1.472 (0.828–2.617)	0.188
Dependent status	2.892 (1.141–7.333)	0.025	2.611 (1.024–6.657)	0.044
Diabetes	1.488 (0.851–2.604)	0.164	1.485 (0.849–2.597)	0.166
COPD	1.585 (0.821–3.060)	0.170	1.563 (0.808–3.022)	0.185
CHF	1.544 (0.604–3.945)	0.364	1.492 (0.582–3.825)	0.405
HTN	1.304 (0.822–2.067)	0.260	1.313 (0.827–2.082)	0.248
Dialysis	5.222 (1.168–23.352)	0.031	3.957 (0.860–18.209)	0.077
ASA 3/4/5	2.365 (1.079–5.185)	0.032	2.314 (1.056–5.074)	0.036
Operation time > 300 min	1.279 (0.841–1.943)	0.249	1.254 (0.825–1.908)	0.290
Albumin less than 3.5 gm/dL	1.830 (1.147–2.918)	0.011	1.742 (1.071–2.831)	0.025

## Data Availability

The American College of Surgeons National Surgical Quality Improvement Program database was used to conduct this study. It is available to all participating institutions. The American College of Surgeons National Surgical Quality Improvement Program and the hospitals participating in the ACS NSQIP are the source of the data used herein; they have not verified and are not responsible for the statistical validity of the data analysis or the conclusions derived by the authors.
